# Parasites as biological tags to assess host population structure: Guidelines, recent genetic advances and comments on a holistic approach^☆^

**DOI:** 10.1016/j.ijppaw.2013.11.001

**Published:** 2013-12-12

**Authors:** Sarah R. Catalano, Ian D. Whittington, Stephen C. Donnellan, Bronwyn M. Gillanders

**Affiliations:** a Marine Parasitology Laboratory, School of Earth and Environmental Sciences, University of Adelaide, Adelaide, SA 5005, Australia; b Southern Seas Ecology Laboratories, University of Adelaide, Adelaide, SA 5005, Australia; c Australian Centre for Evolutionary Biology and Biodiversity, University of Adelaide, Adelaide, SA 5005, Australia; d Parasitology Section, South Australian Museum, Adelaide, SA 5000, Australia; e Evolutionary Biology Unit, South Australian Museum, Adelaide, SA 5000, Australia; f Environment Institute, University of Adelaide, Adelaide, SA 5005, Australia

**Keywords:** Marine parasites, Host stock discrimination, Multidiscipline approach, Next-generation sequencing, Tag guidelines, Dicyemida

## Abstract

We review the use of parasites as biological tags of marine fishes and cephalopods in host population structure studies. The majority of the work published has focused on marine fish and either single parasite species or more recently, whole parasite assemblages, as biological tags. There is representation of host organisms and parasites from a diverse range of taxonomic groups, although focus has primarily been on host species of commercial importance. In contrast, few studies have used parasites as tags to assess cephalopod population structure, even though records of parasites infecting cephalopods are well-documented. Squid species are the only cephalopod hosts for which parasites as biological tags have been applied, with anisakid nematode larvae and metacestodes being the parasite taxa most frequently used. Following a brief insight into the importance of accurate parasite identification, the population studies that have used parasites as biological tags for marine fishes and cephalopods are reviewed, including comments on the dicyemid mesozoans. The advancement of molecular genetic techniques is discussed in regards to the new ways parasite genetic data can be incorporated into population structure studies, alongside host population genetic analyses, followed by an update on the guidelines for selecting a parasite species as a reliable tag candidate. As multiple techniques and methods can be used to assess the population structure of marine organisms (e.g. artificial tags, phenotypic characters, biometrics, life history, genetics, otolith microchemistry and parasitological data), we conclude by commenting on a holistic approach to allow for a deeper insight into population structuring.

## Highlights

• Parasites as biological tags to assess host population structure.
• Recent molecular advances support incorporation of parasite genetic data.
• Guidelines for selection of a parasite species as a tag candidate updated.
• Holistic approach allows for robustness and support of observed result.

## Graphical abstract

Hundreds of dicyemid parasites (white, fuzzy strands) attached to the renal appendage (in red) of a cuttlefish individual.

## 1 Introduction

Determination of the biological identity of a population of marine organisms (for this review, limited to fishes and cephalopods), in relation to neighbouring populations of the same species, is a vital prerequisite in studying the biology, dynamics, interactions and ecological consequences of exploitation on that population (MacKenzie and Abaunza, 1998). This is particularly important given the rise in global fisheries as more species are being targeted and commercially exploited to keep up with increases in demand (Pierce and Guerra, 1994; Evans and Grainger, 2002). Marine species considered at risk as a result of overfishing, evident from declines in biomass and abundance, emphasise the importance of understanding the structure of populations across geographical distributions (Melendy et al., 2005; McClelland and Melendy, 2007). As alluded to already, before a stock can be efficiently managed and policies implemented for future sustainability, the stock needs to be correctly identified (Oliva and Sanchez, 2005).

Many techniques have been used to identify and discriminate stocks, including the application of artificial tags, such as acoustic tags, coded wire tags, passive integrated transponder tags and archival tags. Artificial tags are generally suitable for many species and sizes of organisms, with an added advantage of enabling discrete recognition of each tagged individual (Gillanders, 2009). However, they can be limited in signal detection range and retention over long term studies, with further uncertainties about the influence of the tag on the organism’s behaviour and survivorship (Moser, 1991; Mosquera et al., 2003; Gillanders, 2009). Natural tags, including phenotypic characters (meristic, morphometric and life history traits), otolith chemistry, molecular genetic host markers and parasites, have also been used in population structure studies. In particular, parasites as biological tags have gained wide acceptance in recent decades (MacKenzie, 2002; Poulin and Kamiya, in press), as they can provide a reliable guide to understanding the biology of their host (Pascual and Hochberg, 1996). This is not to say parasites as tags are superior to other methods, but it is recognised that they have helped answer questions on host diet and feeding behaviour, movements and ranges, connectivity of stocks, recruitment patterns of juveniles and phylogenies (Sindermann, 1961; Moser, 1991; Williams et al., 1992; Criscione et al., 2006). Parasites have also been used as bio-indicators of pollution (Poulin, 1992; MacKenzie et al., 1995; MacKenzie, 1999a), and in population studies to discriminate stocks (MacKenzie, 1987, 2002; Lester, 1990; MacKenzie and Abaunza, 1998; Mosquera et al., 2003). Research on parasites as biological tags for marine organisms has increased at a steady rate, with nine papers on this subject published from the 1950s, more than 30 from the 1960s, more than 50 from the 1970s and more than 140 from the 1980s (Williams et al., 1992). Here, we focus on the use of parasites as biological tags for host population discrimination. We use the words ‘stock’ and ‘population’ interchangeably in this review, following the definition provided by Charters et al. (2010) of ‘a spatially distinct group of marine organisms which exhibit no significant mixing with neighbouring individuals’. In agreement with Lester and MacKenzie (2009), we recognise the idea that this distinct group is essentially self-reproducing.

This review begins by briefly commenting on the importance of accurate parasite identification, followed by a summary of the use of parasites as biological tags in population structure studies of fishes and cephalopods. Due to the advent of molecular genetic technologies, the potential to incorporate genetic analyses of parasite population structure alongside genetic analyses of their host is discussed. An updated list of guidelines for selecting a parasite species as an adequate tag candidate is presented, and we conclude by highlighting the benefits of a multidisciplinary approach when investigating the population structure of marine organisms.

## 2 Parasite identification

Along with the need to correctly identify a stock before it can be appropriately managed, parasites also need to be correctly identified before they can be applied as biological tags. We add the caveat that in some cases the minimum necessary identification would be to discriminate each of the parasite species present without the further and potentially time consuming requirement of assigning scientific names. Classical methods commonly used for parasite taxonomic identification involve examining and measuring morphological character traits and using taxonomic keys to define a particular family, genus or species (Baldwin et al., 2012). Although widely used and relatively inexpensive, this form of identification can be difficult for larval stages and further hindered by poor specimen quality and taxonomic uncertainty in the literature. “Species” that exhibit a high level of morphological plasticity also pose a problem (Poulin and Morand, 2000). On one hand, several distinct species may be mistakenly identified as one, or a single morphologically plastic taxon may be interpreted as a species complex inferring significant host population structure.

Another approach to identify parasite species is to use molecular genetic methods (McManus and Bowles, 1996). Indeed, once a sound molecular genetic framework has been established for the species concerned, then higher throughput bar-coding can be applied to much larger sample sets. Another advantage of this approach would be that all stages of the parasite life cycle that could be sampled can be included, potentially increasing the matching parasite data for a larger number of host individuals collected over a longer period of the year. A combination of morphological and molecular genetic methods may therefore be more robust for identifying and discriminating parasite taxa, and should be considered in future studies using parasites as biological tags.

## 3 Parasites as biological tags in population studies of fishes

The two earliest records describing the application of parasites as biological tags in population studies of fishes are that of Dogiel and Bychovsky (1939), who distinguished between groups of sturgeon (*Acipenser* spp.) in the Caspian Sea using the monogenean parasites *Diclybothrium circularis* and *Nitzschia sturionis*, and Herrington et al. (1939), who examined redfish (*Sebastes marinus*) in the Gulf of Maine and suggested the existence of separate populations based on variations in infection levels of the parasitic copepod *Sphyrion lumpi*. Since these investigations over 70 years ago, the use of parasites as biological tags in population structure studies has flourished to include a wide range of fish species and geographical localities. Investigations have primarily focused on, although not limited to, fish species of economic importance, such as herring (e.g. Sindermann, 1961; Parsons and Hodder, 1971; Arthur and Arai, 1980; Moser and Hsieh, 1992), hake (e.g. MacKenzie and Longshaw, 1995; George-Nascimento, 1996; Mattiucci et al., 2004; Sardella and Timi, 2004), cod (e.g. Hemmingsen and MacKenzie, 2001; McClelland and Melendy, 2011), rockfish (e.g. Stanley et al., 1992; Moles et al., 1998; Oliva and Gonzalez, 2004) and hoki (e.g. MacKenzie et al., 2013). A diverse range of taxonomic groups of parasites have also been applied as biological tags (see Table 1 in Williams et al., 1992). In particular, parasites have been used for discovering multiple species in supposedly single species fisheries (e.g. Smith et al., 1981; George-Nascimento, 1996), for discriminating stocks within single species fisheries (e.g. Hemmingsen et al., 1991; Braicovich and Timi, 2008; Henriquez et al., 2011) and for recognising single stocks from multiple breeding populations (e.g. Moser and Hsieh, 1992). Recently, Poulin and Kamiya (in press) performed a meta-analysis to examine the discriminatory power of using parasites to discriminate fish stocks, and found that overall, the probability of correct classification of fish to their group of origin based on parasite data was double that expected by chance alone, supporting the use of parasites as biological tags.

The benefits and limitations of using parasites as biological tags has been extensively reported by Sindermann (1961, 1983), Gibson (1972), MacKenzie (1987, 1999b, 2002), Lester (1990), Moser (1991), Williams et al. (1992), Pascual and Hochberg (1996) and Mosquera et al. (2003), and thus will not be repeated here. The use of parasites as biological tags in population structure studies has also been reviewed by many authors (Sindermann, 1983; MacKenzie, 1987; Lester, 1990; Williams et al., 1992), with a guide to the procedures and methods provided by MacKenzie and Abaunza (1998). The most recent reviews of parasites as biological tags in fish population studies are given by MacKenzie (1999b, 2002), Mosquera et al. (2003) and MacKenzie and Abaunza (2005). In the past 5 years, numerous studies have been published which used parasites as biological tags as the sole approach to discriminate fish stocks (for example Santos et al., 2009; Timi and Lanfranchi, 2009; Timi et al., 2009; Charters et al., 2010; Luque et al., 2010; Mele et al., 2010; Sequeira et al., 2010; Chou et al., 2011; Garcia et al., 2011; Henriquez et al., 2011; Hutson et al., 2011; Khan et al., 2011; McClelland and Melendy, 2011; Moore et al., 2011; Braicovich et al., 2012; Reed et al., 2012; Costa et al., 2013; MacKenzie et al., 2013; Oliva, 2013).

With this increase in the number of studies using parasites as biological tags to discriminate host stocks, Lester and MacKenzie (2009) provide a word of caution. They highlight that although differences in parasite fauna may be observed from fish collected at different geographical localities, it does not necessarily mean that there are multiple fish stocks, as many parasites are transient and may only be present sometimes. Leading on from this, it is suggested that a deeper insight or more robust conclusions may be gained from using a multidisciplinary approach to determine stock structure, a topic that will be discussed further in Section 7.

## 4 Parasites as biological tags in population studies of cephalopods, including comments on the dicyemid mesozoans

Over the last 20 years, the value of cephalopods in international commercial fisheries has increased rapidly (Pierce and Guerra, 1994; Pascual et al., 1996). However, cephalopods are highly susceptible to overfishing with little opportunity for recovery, owing to their short life spans, variable growth rates and semelparous breeding strategies (Pierce and Guerra, 1994; Boyle and Boletzky, 1996). Therefore, it is important to be able to recognise stock boundaries to ensure management policies governing commercial cephalopod fisheries are well-informed.

While numerous parasite species from a range of taxonomic groups have been described from cephalopods (Hochberg, 1983, 1990), their application as biological tags in population studies is rare (Pascual and Hochberg, 1996). The first study where parasites were used to examine cephalopod stock structure was performed by Smith et al. (1981). The authors used a multidisciplinary approach of allozyme electrophoresis, host morphology and prevalence of parasites in arrow squid *Nototodarus sloani* from New Zealand waters to assess stock structure, with the combined results supporting the occurrence of two species of arrow squid in these waters. It is doubtful whether the same result would have been concluded if parasites alone were used, as one parasite species did not support stock separation whereas the other did. A few years later, Dawe et al. (1984) addressed the issue of stock discrimination in the short-finned squid *Illex illecebrosus*, also employing a multidisciplinary approach by comparing data on host size, maturity, distribution of early life history stages and incidence of certain parasites. However the parasites examined were of little use as biological tags, as they had a broad geographic distribution, were generalist rather than specialist parasites, and could not be identified to species. Later, Bower and Margolis (1991) and Nagasawa et al. (1998) examined the helminth parasites of the flying squid, *Ommastrephes bartrami*, in the North Pacific Ocean. Bower and Margolis (1991) suggested that parasites may be useful tools in determining the stock structure of the flying squid, and Nagasawa et al. (1998) statistically tested parasite intensity of infection among collection localities to lend support to the occurrence of four flying squid stocks in these waters. The most recent study that has used parasites of cephalopods as biological tags is by González and Kroeck (2000). They studied the parasite fauna composition of shortfin squid *Illex argentinus* in San Matías Gulf, southwest Atlantic, with differences in composition, prevalence and mean intensity of enteric parasites between localities lending support to stock structuring.

An additional group of parasites, dicyemid mesozoans, have been suggested as potential tag candidates to help discriminate cephalopod stock structure (Hochberg, 1990; Catalano, 2013). These parasites are simple in morphology, highly host-species specific and found with high intensity in the renal appendages of almost all benthic cephalopod species examined to date (Furuya, 1999; Furuya et al., 2004). The use of dicyemid parasites as biological tags for cephalopod stock discrimination has been tested, with significant difference in dicyemid fauna composition between cephalopod species, and among cephalopod individuals of the same species collected from different geographical localities (Catalano et al., unpublished). However it must be highlighted that confusion exists in the literature on the validity of certain taxa within this phylum along with the morphological traits used to delineate species boundaries (Catalano, 2012). Nonetheless, by incorporating a molecular genetic framework, and comparing results between dicyemid parasite and host genetic analyses, this approach may still prove valuable in assessing cephalopod population structure beyond any single approach.

## 5 Recent genetic advances

Beverley-Burton (1978) was the first to use genetic analyses of parasite populations as a tool for host stock identification. The frequencies of different acid phosphatase allozymes in the larval nematode *Anisakis simplex* suggested that there may be two distinct groups of Atlantic salmon in the Atlantic Ocean. Other authors have used genetic methods (multilocus allozyme electrophoresis) to identify *Anisakis* larvae to species level, then by evaluating the relative proportions of these nematodes across sampling localities, recognised multiple discrete host stocks (Mattiucci et al., 2004, 2008).

In recent years there have been major technological advances in the field of molecular genetics, providing the ability to sequence multiple markers or whole genomes in a short time span with low costs, e.g. next generation sequencing (Schuster, 2007; Mardis, 2008; Quail et al., 2012). In fisheries science, multiple molecular markers such as allozymes, mitochondrial DNA, microsatellite and minisatellite loci, random amplified polymorphic DNA (RAPD) and single nucleotide polymorphisms (SNPs), have all been used to analyse stock structure of marine organisms directly (Carvalho and Hauser, 1994; Thorpe et al., 2000; Baldwin et al., 2012; Ovenden et al., 2013). This has proven useful for deep-sea species where tag-recapture techniques are difficult to apply (e.g. Roques et al., 2002; Friess and Sedberry, 2011; Varela et al., 2013). As candidate molecular markers that are under selection can now be identified and genotyped readily, the capability also exists to assess genetic differences between recently diverged groups or between incompletely isolated groups, which was otherwise problematic with neutral molecular markers (Swain et al., 2005; Lamichhaney et al., 2012). These advances have subsequently helped to unveil previously unrecognised patterns of geographic genetic structure in marine organism (Sala-Bozano et al., 2009).

What is yet to be realised in any substantive way is the application of these new molecular genetic technologies, with high throughput and increased resolution, to parasitological studies of host population structure. Additional layers of information may be gained by contrasting the genetic population structure of parasite and host not just for determining host population structure but details of the hosts population biology (e.g. Pacific sardines – Baldwin et al. 2012). In the one example that we can find where the same class of high resolution population markers (microsatellites) were used in both parasite and host, Criscione et al. (2006) found that trematode parasite genetic structure identified source populations of host steelhead trout (*Oncorhynchus mykiss*) with four times more accuracy than the host’s own genotype. This finding highlights how differences in host and parasite environmental tolerances, population size and connectivity influence their rates of population differentiation. We are not suggesting skipping over host genetics to assess population structure, but instead advocating the inclusion of parasite genetics into these studies for what may provide an additional line of confirmatory evidence or new insights into host structuring. As with any molecular genetic study, it is important to recognise that the results will depend on, and may differ, with the type of molecular genetic markers used, the number of loci examined, the geographical scope of the study, the number of fish sampled and the population biology of the parasite taxa examined (Baldwin et al., 2012). To boost the analytical power of these genetic analyses, it is recommended to increase the sample size, the number of molecular markers and loci used, and the number of parasite taxa included (Ovenden et al., 2013).

## 6 Guidelines for selecting an ideal parasite species as a tag candidate

According to Thorrold et al. (2002), the properties of a general tag, including artificial and environmental tags, genetic markers and parasites, should have the following characteristics:

1) Retention of the tag over an appropriate length of time.
2) No effect of the tag on the marked organism (invisible to predators, non-toxic, no effect on growth or survival).
3) Ability to mark a large number of individuals in a cost-effective manner.
4) Be relatively quick and inexpensive to detect.

In addition to these characteristics for tags in general, there are also a number of specific guidelines presented in the literature which highlight the desirable requirements a parasite species should have in being considered as a biological tag candidate. For example, the following are taken from Sindermann (1961, 1983), MacKenzie (1987, 1999b), Lester (1990), Moser (1991), Williams et al. (1992) and MacKenzie and Abaunza (1998):

1) The parasite species should have different levels of infection in the host at different geographical locations.
2) The life cycle of the parasite species should preferably involve only a single host as more information is needed on the biotic and abiotic factors influencing transmission between hosts for those parasite species with multi-host life cycles.
3) The life span of the parasite species in the host needs to cover the duration of the investigation as a minimum.
4) The prevalence of the parasite species should remain relatively stable between seasons and years.
5) The parasite species should be easily detected, preferably by gross examination.
6) The parasite species should have no effects on the behaviour or survival of the host.

It is wise to acknowledge that these guidelines are just that, recommendations rather than set rules. A single parasite species would rarely have all of these attributes, so compromises usually have to be made (Sindermann, 1983; MacKenzie and Abaunza, 1998). For instance, in some cases, anisakids and trypanorhynchs, parasites that require at least three host species to complete their life cycle (contravening guideline 2 above), have been found to be the best tag candidates (Boje et al., 1997; MacKenzie and Abaunza, 1998; Timi, 2007; Chou et al., 2011). Additionally, the use of several different parasites and even whole parasite assemblages as tags may be more reliable than using a single species, as a greater number of the guidelines may be met to yield a more complete assessment of host population structure (Timi, 2003, 2007; Sardella and Timi, 2004; MacKenzie and Abaunza, 2005). Note that if this approach is selected, only parasite species highlighted as permanent (recognisable for most of the life of the host) should be considered (Lester and MacKenzie, 2009).

## 7 Holistic approach to discriminate population structure of marine organisms

Rather than focusing on only a single approach to discriminate fish stocks, it may be of greater benefit to consider incorporating data across disciplines, as different stock identification approaches have different levels of sensitivities (Waldman, 2005). Meristics, parasite data and microsatellite markers can be used to detect differences that have arisen in the recent past, whereas other techniques which are more conservative, such as allozymes and coding DNA, require longer periods of isolation for differences to become recognisable (Cadrin, 2011). Therefore by combining approaches across disciplines, a more robust baseline is created and greater confidence in the observed result is gained (Cadrin, 2010). For example, Zischke et al. (2013) used morphometric measurements of 12 fixed anatomical characters and variation in parasite abundance of seven species to examine the stock structure of wahoo *Acanthocybium solandri* collected in three regions, with the results from both analyses complementing one another in stock boundary estimates. For future studies examining the global stock structure of wahoo, they suggest incorporating additional techniques such as otolith microchemistry and genetic microsatellites. In another study, life history data (age at first maturity, size structure and growth patterns), otolith microchemistry and parasite fauna composition were used to distinguish stocks of southern blue whiting *Micromesistius australis* between two main spawning grounds in the southwest Atlantic Ocean and southeast Pacific Ocean (Arkhipkin et al., 2009; Niklitschek et al., 2010). This contrasted with the results of earlier genetic studies based on mitochondrial DNA haplotype frequencies, which did not detect any significant differences between these areas (Shaw, 2003, 2005). More recently, Baldwin et al. (2012) reviewed the use of fish morphometrics, artificial tags, fish genetics, parasite genetics and parasites as biological tags to identify subpopulations of marine fishes and affirmed the merits of a holistic approach, integrating data from fish and parasite based techniques (both community and genetic), to resolve stock structuring. Other authors also express support for using complementary methods from a broad spectrum, with different ecological and evolutionary characteristics, to provide a comprehensive picture of the population structure of marine organisms (Begg and Waldman, 1999; Cadrin et al., 2005; Sala-Bozano et al., 2009).

A multidisciplinary approach also provides additional techniques available for use in subsequent stock analysis studies (Cadrin, 2011). For example, Roques et al. (2002) used eight microsatellite loci initially to identify fish stocks of the western group of deepwater redfish *Sebastes marinum*, with these stocks subdivided into four smaller groups in a subsequent study that used parasite species prevalence data (Marcogliese et al., 2003). Early research on the winter flounder stock structure primarily focused on migration, life history rates and analysis of meristic characters, however over time, genetic analyses, parasite fauna composition, modelling analyses, otolith chemistry and telemetry tagging were incorporated, building on from this initial framework for a more robust and supported insight (Cadrin, 2011). Stock delineation of the Pacific hake *Merluccius productus* has also been assessed with a variety of techniques in different studies, including parasite analyses (Kabata and Whitaker, 1981), otolith morphology (McFarlane and Beamish, 1985), biological parameter estimates (Beamish and McFarlane, 1985; King and McFarlane, 2006) and mitochondrial sequence data (King et al., 2012). In summary, it seems viable that a multidisciplinary approach which integrates data across fields, such as molecular genetics, biometrics, life histories, modelling, otolith microchemistry analyses, artificial tagging studies and parasitological surveys, may provide a deeper and more robust insight into the population structuring of marine organisms in contrast to studies using a sole approach. There is also a need to utilise the recent advancements in these fields as tools to improve our understanding of stock boundaries.

## 8 Closing remarks

Our review highlights the usefulness of parasites as biological tags in population structure studies of marine organisms. However caution must be taken in selecting the most appropriate tag candidate species, as well as considerations on the number of taxa to include, the method of parasite identification and the way the data are analysed. With the recent advancement of molecular genetic techniques, we highlight the potential to include parasite genetic data alongside host intra-specific molecular genetic data, an area that is currently under-exploited. In particular the use of high resolution neutral markers or loci under selection in both the parasite and host to detect recent demographic driven host population structure is unexplored. As multiple approaches can be used to assess population structure of marine organisms, each with their own benefits and limitations, we ultimately advocate the integration of data from multiple disciplines for a deeper insight into population structuring. Due to the different levels of sensitivity of each method, additional layers of information may be gained or weak inferences may be better assessed using a holistic approach.
